# Influence of Temperature during Freeze-Drying Process on the Viability of *Bifidobacterium longum* BB68S

**DOI:** 10.3390/microorganisms11010181

**Published:** 2023-01-11

**Authors:** Yue Sang, Jian Wang, Yongxiang Zhang, Haina Gao, Shaoyang Ge, Haihong Feng, Yan Zhang, Fazheng Ren, Pengcheng Wen, Ran Wang

**Affiliations:** 1 College of Food Science and Engineering, Gansu Agricultural University, Lanzhou 730070, China; 2 Key Laboratory of Functional Dairy, Co-Constructed by Ministry of Education and Beijing Government, Department of Nutrition and Health, China Agricultural University, Beijing 100083, China; 3 School of Food and Health, Beijing Technology and Business University, Beijing 100048, China; 4 Hebei Engineering Research Center of Animal Product, Sanhe 065200, China

**Keywords:** freeze-drying, pre-freezing temperature, sublimation temperature, *Bifidobacterium longum*

## Abstract

Maintaining optimum temperature during freeze-drying is crucial to ensuring the viability of strains. In this study, we evaluated the effect of pre-freezing, sublimation and desorption temperatures on the viability of *Bifidobacterium longum* BB68S (BB68S). Moreover, we examined the water content, water activity, enzyme activities, and scanning electron microscope of BB68S to explore mechanisms underpinning the effect of temperature on viability. Our analyses revealed the highest survival rates of BB68S collected after pre-freezing and sublimation drying at −40 °C (94.9 ± 2.2%) and −10 °C (65.4 ± 3.8%), respectively. Additionally, response surface methodology demonstrated that the optimum conditions for freeze-drying of BB68S were pre-freezing temperature at −45.52 °C and sublimation temperature at −6.58 °C, and the verification test showed that survival rates of BB68S could reach 69.2 ± 3.8%. Most of the vitality loss occurred during the sublimation drying phase. Further studies showed that different sublimation temperatures affected water content and activity, β-galactosidase, lactate dehydrogenase, Na^+^-K^+^-ATP and Ca^2+^-Mg^2+^-ATP activities. In conclusion, the temperature during freeze-drying, especially sublimation temperature, is a key factor affecting the survival rate of BB68S, and the vitality loss during freeze-drying process might be due to compromised cell membrane integrity and permeability.

## 1. Introduction

Probiotics play a key role in the food and dairy industries, and probiotics have been shown to exert positive effects on health status of the host [1]. Powdered probiotics are convenient for subsequent processing and transportation, and have longer shelf lives compared to those of liquid probiotics [2,3,4]. Freeze-drying achieves better survival rates compared to other drying techniques, thus making it an effective technique for the production of probiotic powders [5]. However, during the freeze-drying process, long-term exposure to extreme environmental conditions could damage the physical properties of cell membranes or the structure of cell-sensitive proteins, thus lowering the survival rates of freeze-dried probiotics [6,7]. Recently, it was demonstrated that optimization of conditions during the freeze-drying process improves the survival rates of probiotics [8,9,10].

The freeze-drying process has three stages: pre-freezing, sublimation drying and desorption drying [11]. The water contained in the sample converts to ice crystals during the pre-freezing stage. This is followed by sublimation drying, where the ice crystals undergo sublimation. Finally, the water molecules bound to the samples are released during the desorption drying stage [11]. Wang et al. [12] demonstrated that pre-freezing temperature could influence the survival rates of different *Lactobacillus plantarum* strains. Aragón-Rojas et al. [13] observed that sublimation temperatures could affect the survival rates of *Lactobacillus fermentum* K73. The temperature during different freeze-drying stages might affect the survival rates of probiotic strains. It is crucial to optimize pre-freezing, sublimation and desorption temperatures for the improvement of survival rates. However, the mechanisms underpinning the effect of freeze-drying temperatures on the viability of bacterial strains have remained unexplored.

At present, numerous species of *Bifidobacterium* are used as probiotics, among which *Bifidobacterium longum* is widely used and has a history of safe use [14]. *Bifidobacterium longum* BB68S (CGMCC No. 14168, BB68S) was isolated from healthy centenarians in Bama longevity villages of Guangxi province in China. The aim of this investigation was to optimize pre-freezing, sublimation and desorption temperatures during the freeze-drying process of BB68S. Furthermore, we explore the putative mechanisms through which freeze-drying temperatures affect bacterial survival rate to provide the rationale behind the selection of temperatures during the freeze-drying of probiotics.

## 2. Materials and Methods

### 2.1. Bacterial Strains and Culture Conditions

BB68S was cultured in Man–Rogosa–Sharpe broth at 37 °C for approximately 12 h. The biomass fermentation was performed in a 5 L fermenter (BLBIO-5GJ-2-H, Shanghai Bailun Biotechnology Co., Ltd., Shanghai, China) with 3 L working volume, at 80 rpm for 12 h. The medium composition was 1.5% yeast peptone, 1.0% yeast extract powder, 0.5% beef extract powder, and 4.0% glucose. The microbial inoculation rate was 2% (*v*/*v*).

### 2.2. Freeze-Drying Temperature Optimization

Pre-freezing survival rates of BB68S were calculated after 2 h of pre-freezing at different temperatures (−20 °C, −40 °C and −60 °C). Sublimation drying survival rates of BB68S were calculated after 2 h of pre-freezing (−40 °C) and 20 h of sublimating at different temperatures (−20 °C, −40 °C and −60 °C). Desorption drying survival rates of BB68S were calculated after 2 h of pre-freezing (−40 °C), 20 h of sublimating (−10 °C), and 15 h of treating with different desorption temperatures (20 °C, 25 °C and 30 °C).

### 2.3. Response Surface Methodology (RSM) Optimization

Design Expert Version 12.0 (Stat-Ease Inc., Minneapolis, MN, USA) software was applied for RSM design and statistical analysis. According to RSM, an experimental design 3^2^ was applied to optimize pre-freezing temperature (−20 °C, −40 °C and −60 °C) and sublimation temperature (−20 °C, −10 °C and 0 °C) during the freeze-drying process (Table 1). The desorption temperature was 25 °C. The survival rates of BB68S (Y) were used as response values to obtain the optimum pre-freezing temperature (X_1_) and sublimation temperature (X_2_). The relationship between response (Y) and factors (X) was described using the following equation:

$$Y=\beta_{0}\;+\sum_{i=1}^{k}\beta_{i}X_{i}+\sum_{i=1}^{k}\beta_{\mathrm{ii}}X_{i}^{2}+\sum_{i=1}^{k}\sum_{j=i+1}^{k-1}\beta_{\mathrm{ij}}X_{i}X_{j}$$

In this equation, Y represents the response variable (survival rate); x_i_ and x_j_ represent independent coded variables (X_1_, pre-freezing temperature, X_2_, sublimation temperature); β_0_, β_i_, β_ii_ and β_ij_ represent the coefficients for intercept, linear, quadratic and interaction, respectively.

### 2.4. Survival Rate Measurements

Viable cell counts were detected using the plate-counting method. The powder was rehydrated with PBS and incubated on MRS plates at 37 °C for 48 h for counting. The survival rates (%) after pre-freezing, after drying and after freeze-drying were calculated as follows: N1/N0 × 100%, N2/N0 × 100% and N3/N0 × 100%, where N1 represents the viable counts after pre-freezing, N2 represents the viable counts after drying, N3 represents the viable counts after freeze-drying, and N0 represents the viable counts before freeze-drying [12].

### 2.5. Water Content and Water Activity (a_w_) Analysis

The a_w_ was measured using a water activity meter (Novasin AG, Lab Master-aw, Lachen, Switzerland) at room temperature. The freeze-dried samples were oven dried at 105 °C until constant weight. The weight loss was measured to estimate water content.

### 2.6. Preparation of Cell-Free Extracts and Extracellular Supernatants

After rehydration, the bacterial cells were centrifuged at 10,000× *g* for 10 min at 4 °C The supernatant was used to measure extracellular enzyme activity. For collecting cell-free extracts, 10 mg/mL lysozyme was added, and the mixture was kept at 37 °C for 15 min. Then, 4 mol/L sodium chloride solution was added, and incubated at 37 °C for 50 min. Finally, the mixture was centrifuged at 10,000× *g* for 10 min.

### 2.7. β-Galactosidase, Lactate Dehydrogenase, Na^+^-K^+^-ATP and Ca^2+^-Mg^2+^-ATP Activity Analysis

The activities of β-galactosidase, lactate dehydrogenase, Na^+^-K^+^-ATP and Ca^2+^-Mg^2+^-ATP were analyzed using commercial kits (Beijing Solaibao Technology Co., Ltd., Beijing, China) according to the instructions. One unit of β-galactosidase activity is defined as the amount of enzyme that releases 1 nmol o-nitrophenol per hour at 37 °C. The amount of released o-nitrophenol was measured at 400 nm. One unit of lactate dehydrogenase activity is defined as the amount of enzyme that releases 1 nmol pyruvate per min at 37 °C. The amount of pyruvate was measured at 450 nm. One unit of Na^+^-K^+^-ATPase and Ca^2+^-Mg^2+^-ATPase are both expressed as 1 μmol of inorganic phosphate released per milligram of protein per hour, and the sample was measured at 660 nm.

### 2.8. Scanning Electron Microscopy (SEM)

Leyophilized powder was fixed on a glass slide usingconductive tape and sputtered with gold at 10 mA for 2 min. The morphology of gold-clad samples was evaluated with a Hitachi SU8020 field emission scanning electron microscope (Japan) at 5 KV.

### 2.9. Statistical Analysis

All experiments were conducted with three biological replicates, and the data are expressed as the mean ± standard deviation (SD). Statistical analyses were performed using SPSS (IBM Corp., Armonk, NY, USA). Significant differences among the data were detected using ANOVA. *p* < 0.05 was used as a threshold for statistically significant differences.

## 3. Results

### 3.1. Survival Rates of BB68S under Different Temperatures during Freeze-Drying Process

**Figure 1:**
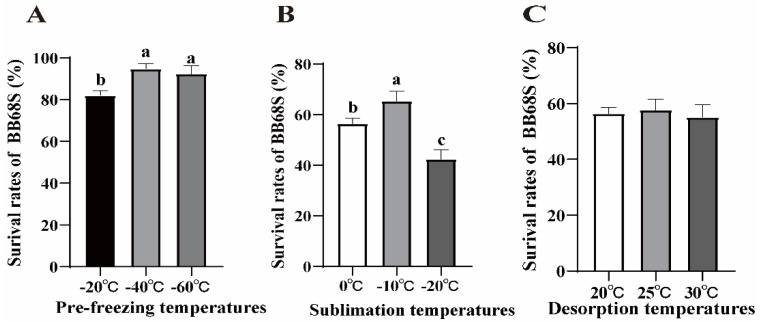
Influence of different temperatures during the freeze-drying process on the survival rate of Bifidobacterium longum BB68S. (**A**) Pre-freezing survival rates of BB68S under different pre-freezing temperatures; (**B**) Sublimation drying survival rates of BB68S under different sublimation temperatures; (**C**) Desorption drying survival rates of BB68S under different desorption temperatures. Different letters indicate significant differences (*p* < 0.05).

The BB68S obtained by fermentation was freeze-dried under different temperatures, and survival rates of samples collected after pre-freezing, sublimation drying and desorption drying were calculated (Figure 1A–C). The survival rates of BB68S collected after pre-freezing at −40 °C were higher (*p* < 0.05) (94.9 ± 2.2%) compared to those after pre-freezing at −20 °C (82.1 ± 2.2%) and −60 °C (92.3 ± 3.8%) (Figure 1A). The survival rates of BB68S collected after sublimation drying at −10 °C were higher (*p* < 0.05) (65.4 ± 3.8%) compared to those collected after sublimation drying at −20 °C (42.3 ± 3.8%) and 0 °C (56.4 ± 2.2%) (Figure 1B). No differences in survival rates were observed among samples collected after desorption drying at 20 °C, 25 °C and 30 °C (Figure 1C). Overall, different pre-freezing temperatures and sublimation temperatures could affect survival rates of BB68S; however, most of the vitality loss occurred during the sublimation drying phase, and this result was consistent with previous studies [12]. Altogether, these data indicate that optimizing sublimation conditions could be the most efficient strategy for improving the viability of freeze-dried BB68S.

### 3.2. Response Surface Methodology Optimization of Pre-Freezing Temperatures and Sublimation Temperatures

The effect of pre-freezing temperatures (X_1_) and sublimation temperatures (X_2_) on the survival rate of BB68S was evaluated by the application of RSM, and predicted values were similar to experimental values (Table 1). Based on the experimental data, the predicted response Y could be expressed using the following equation:

Y = 55.28 − 5.85 X_1_ + 7.63 X_2_ − 2.92 X_1_* X_2_ − 12.40 X_1_^2^ − 12.34 X_2_^2^

where Y represents the survival rate, and X_1_ and X_2_ are the pre-freezing temperature and sublimation temperature, respectively.

**Figure 2:**
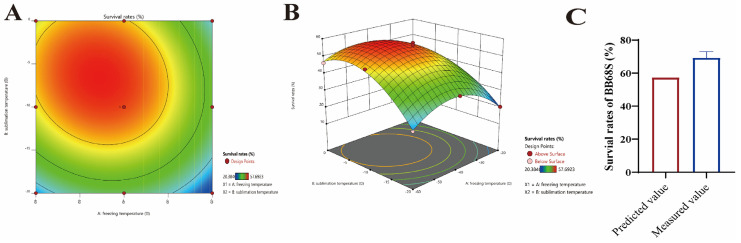
Effects of pre−freezing temperature and sublimation temperature on the survival rates of *Bifidobacterium longum* BB68S after freeze-drying process. (**A**) Contour; (**B**) 3D Surface; (**C**) Predicted values and measured values for survival rates of BB68S under optimal conditions.

As shown in Table 2, the *p*-value of the model was significant (*p* < 0.0001), and the *p*-value for lack of fit was not significant (*p* = 0.837), indicating a good fit. The coefficient of determination (R^2^) was higher than 90% (R^2^ = 0.9899), and adjusted R^2^ was 0.9827, indicating that the model could explain 90% of the response variation. In decreasing order of F values, the factors affecting the survival rates of BB68S were X_1_^2^ (*F*-value = 149.28) > X_2_^2^ (*F*-value = 147.74) > X_2_ (*F*-value = 122.72) > X_1_ (*F*-value = 72.29) > X_1_* X_2_ (*F*-value = 11.96). Figure 2 showed that as the increase of sublimation temperature, survival rates of BB68S first increased and then decreased in the range of pre-freezing temperature (−20~−60 °C). Additionally, in the range of sublimation temperature (−20~−0 °C), survival rates also increased at first and then reduced with the increase of pre-freezing temperature. According to the quadratic polynomial regression model, the optimal pre-freezing temperature was −45.52 °C, and the optimal sublimation temperature was −6.58 °C. Under the optimal conditions, the predicted value for BB68S survival rate was 57.40%. Additionally, the verification test showed that survival rates of BB68S could reach 69.2 ± 3.8% at a pre-freezing temperature of −45.5 °C and sublimation temperature of −6.5 °C.

### 3.3. Water Content, a_w_ Activities of β-Galactosidase, Lactate Dehydrogenase, Na^+^-K^+^-ATP and Ca^2+^-Mg^2+^-ATP, and Scanning Electron Microscope of BB68S under Different Sublimation Temperatures

**Figure 3:**
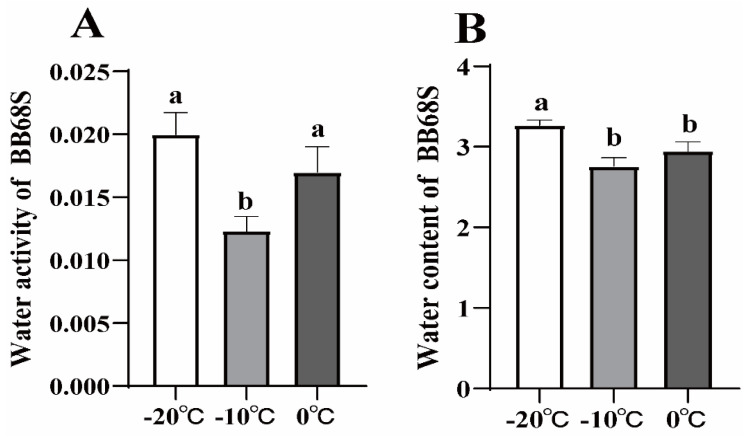
Water content (**A**) and water activity (**B**) of *Bifidobacterium longum* BB68S exposed to various sublimation temperatures. Different letters indicate significant differences (*p* < 0.05).

The water content and a_w_ of BB68S under different sublimation temperatures are shown in Figure 3. The lowest water content and a_w_ of BB68S were obtained under the condition of sublimation temperature of −10 °C (*p* < 0.05).

**Figure 4:**
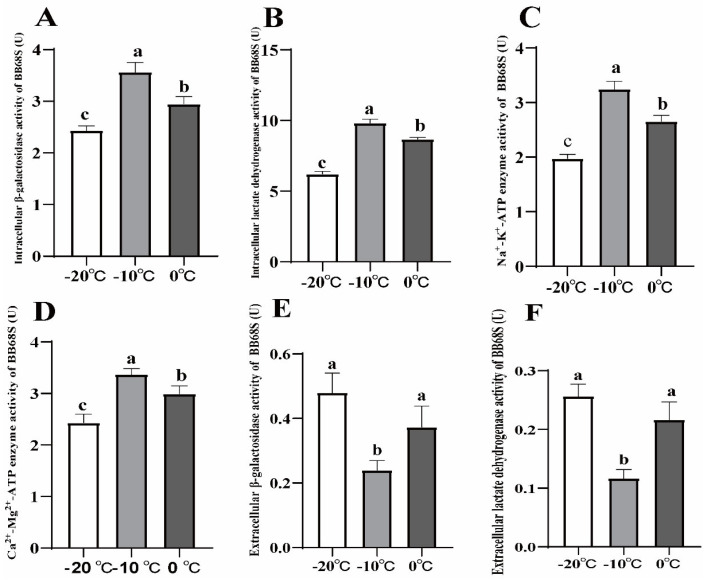
Intracellular β−galactosidase (**A**), intracellular lactate dehydrogenase (**B**), Na^+^-K^+^-ATP (**C**), Ca^2+^-Mg^2+^-ATP (**D**), extracellular β-galactosidase (**E**) and extracellular lactate dehydrogenase (**F**) activities of *Bifidobacterium longum* BB68S exposed to various sublimation temperatures. Different letters indicate significant differences (*p* < 0.05).

As shown in Figure 4, intracellular β-galactosidase, lactate dehydrogenase, Na^+^-K^+^-ATPase and Ca^2+^-Mg^2+^-ATPase activities were significantly higher at the sublimation temperature of −10 °C than those of at 0 °C and −20 °C (*p* < 0.05). As for extracellular β-galactosidase and lactate dehydrogenase, activities revealed that those of the powders obtained at a sublimation temperature of −10 °C were the lowest (*p* < 0.05).

**Figure 5:**
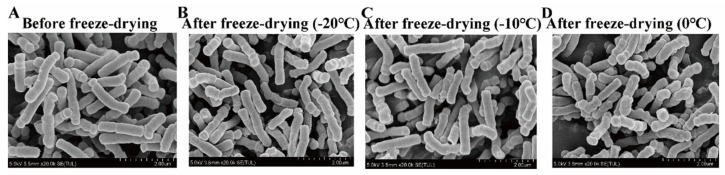
Scanning electron microscope photographs of *Bifidobacterium longum* BB68S exposed to various sublimation temperatures.

The SEM of BB68S obtained under different sublimation temperatures are shown in Figure 5. The intact cells maintained a smooth surface with a typical rod shape, in which most of the cell morphology was intact and full, no deformed cells were found, and there was no adhesion between cells.

## 4. Discussion

In the current study, we evaluated the effect of pre-freezing and sublimation temperatures on the survival rate of BB68S. Previous research showed that pre-freezing temperatures could affect pre-freezing rates, under the same pre-freezing time. The pre-freezing rate showed a trend to reduce with the increase of pre-freezing temperature [15]. On the one hand, a low pre-freezing rate leads to intracellular water flowing into the extracellular environment and the formation of extracellular crystals, thus resulting in the increase of solute levels and imbalance of osmosis. On the other hand, a high pre-freezing rate leads to not enough intracellular water flowing into the extracellular environment, and the formation of too-large intracellular crystals could seriously damage the cell walls of strains [16]. Thus, extreme pre-freezing rates might result in a reduction of survival rates. In the current study, in comparison to −20 °C and −60 °C, −40 °C was considered to be the optimum freezing temperature. Furthermore, in the present study, survival rates of BB68S were affected by different sublimation temperatures. Aragón-Rojas et al. [13] found that cell viability was closely related to sublimation conditions. Increasing sublimation temperatures increased sublimation rates, and decreased the time required for sublimation. The decrease in drying time was beneficial for survival rates of the strain. The enhancement in sublimation time could lead to the removal of bound water, and damage protein structures [17]. In this study, survival rates of BB68S were also higher at sublimation temperature of −10 °C compared to −20 °C. However, when the sublimation temperature was higher than the vitreous transition temperature, the occurrence of collapse phenomenon might negatively affect survival rates [18]. This might explain why BB68S survival rates were higher at sublimation temperature of −10 °C in comparison to 0 °C. After the sublimation drying stage, the desorption of a large amount of remaining unfrozen water in the concentrated solute occurred to reduce water content and a_w_. However, desorption temperature did not seem to affect the survival rates of BB68S in the current study.

RSM is a collection of mathematical and statistical methods, and RSM is often used for the development, improvement and optimization of process parameters [19]. Recently, one method for the optimization of freeze-drying conditions is the application of RSM [20]. Therefore, in the current study, RSM was used to further optimize pre-freezing temperature and sublimation temperature to achieve the improvement of survival rates. An interaction between pre-freezing temperature and sublimation temperature on survival rates could be observed in this study. It has been demonstrated that ice crystals formed in the pre-freezing phase could determine the occurrence of ice crystal removal in the drying phase. Large and contiguous ice crystals are beneficial to vapor migration, and thus ensure fast drying [21]. Extremely high pre-freezing temperature has been demonstrated to result in incomplete freezing, thus leading to the expansion and foaming of the samples in the drying phase [12].

Similar to previous reports, water content was associated with sublimation temperatures during the freeze-drying process [13]. Water content and a_w_ are crucial to the storage of probiotics products [22,23,24]. Therefore, the residual moisture content should be low enough to prevent deterioration during the storage period. In this study, a_w_ of each dried product was in the range of 0.012–0.020, and water content was in the range of 2.76–3.27%, which were equal to or lower than previously reported values, thus the samples were considered sufficiently dehydrated under drying conditions used in this study [22,25]. When a_w_ and water content of obtained probiotics after freeze-drying are in the optimal range, water may interact with functional groups and block reaction sites, thus avoiding interactions with oxygen and oxidation reactions that lead to lipid and protein degradation in cells [24].

ATP-hydrolyzing enzymes such as Na^+^-K^+^-ATPase and Ca^2+^-Mg^2+^-ATPase membrane-bound enzymes, and ATPase are crucial to cell functions and intracellular ions gradients [26,27]. The increased Na^+^-K^+^-ATPase and Ca^2+^-Mg^2+^-ATPase activities of bacterial cells are considered to be related to the enhancement of cell membrane integrity and tolerance to freeze-drying [28]. β-galactosidase relates to the degradation of lactose, and helps probiotics perform their probiotic functions [29]. On the one hand, the activity of β-galactosidase responds to the fermentation capacity of the strain, which breaks down lactose into galactose and glucose. On the other hand, since β-galactosidase is a bacterial endogenous enzyme, which is a macromolecular protein, generally β-galactosidase is released from bacterial extracellular when membrane damage occurs in bacterial cells, and it can be used as an important indicator of changes in microbial cell membrane permeability. Lactate dehydrogenase could reduce pyruvate to lactate during normal fermentation, and its activity reflects the acid production capacity of the strain, which tends to decrease after freeze-drying [30]. The freeze-drying process can disintegrate the membrane and allow the enzyme to escape to the external environment, adversely affecting β-galactosidase and lactate dehydrogenase activities [31]. Therefore, β-galactosidase and lactate dehydrogenase activities could be used to evaluate cell membrane damage. The differences in the key enzyme activities of obtained BB68S under different sublimation temperatures indicated that sublimation temperatures might affect the survival rates of strains by affecting cell membrane integrity and permeability.

During the dehydration of strains, most of the water inside the cells was expelled through membrane channels. During the freeze-drying process, the cells could not fully recover due to the destruction of cell structure by dehydration, thus reducing cell viability [32]. In the present study, no obvious differences were observed in cell morphology of freeze-dried BB68S, and no cells with severe morphological breakage were observed. Considering enzyme activities of BB68S, sublimation temperatures might affect survival rates by affecting cell membrane integrity and permeability. Thus, differences in cell morphology of BB68S through SEM under different sublimation temperatures could not be observed.

## 5. Conclusions

Freeze-drying is an excellent method for preserving probiotics; however, negative effects on the survival rates of strains are inevitable. This experiment showed that pre-freezing temperatures and sublimation temperatures during freeze-drying affects the survival rates of BB68S, and the optimization of pre-freezing and sublimation temperatures could increase the viability of BB68S. Additionally, survival rates affected by different temperatures might be related to cell membrane integrity and permeability. These results provide more strategy for the improvement of probiotics survival rates. Furthermore, this study provides theoretical support for selecting pre-freezing and sublimation temperatures during the freeze-drying process.

## Figures and Tables

**Table 1:** Response surface methodology optimization designs of the effect of pre-freezing temperatures and sublimation temperatures on the survival rates of *Bifidobacterium longum* BB68S.

	Factors		Response Variables	
Run	X_1_ (°C)	X_2_ (°C)	Survival Rate (%)	Predicted Survival Rate (%)
1	−20	−20	20.4 ± 2.4^Op^	19.98
2	−20	−10	36.5 ± 1.2	37.03
3	−20	0	29.5 ± 1.2	29.40
4	−40	−20	35.4 ± 1.8	35.32
5	−40	−10	55.1 ± 2.2	55.28
6	−40	−10	57.7 ± 3.8	55.28
7	−40	−10	53.8 ± 3.8	55.28
8	−40	−10	52.6 ± 4.4	55.28
9	−40	−10	56.4 ± 2.2	55.28
10	−40	0	51.3 ± 2.2	50.57
11	−60	−20	25.4 ± 1.9	25.86
12	−60	−10	50.0 ± 3.8	48.74
13	−60	0	46.2 ± 3.8	46.95

X_1_ = Pre-freezing temperature (°C); X_2_ = Sublimation temperature (°C); Results were expressed as means ± standard deviation.

**Table 2:** Regression coefficients obtained by response surface methodology explain the effects of pre-freezing temperatures and sublimation temperatures on survival rates of *Bifidobacterium longum* BB68S.

Survival Rate	Intercept					R^2^	Adj R^2^	*p*-Value (Model)	*p*-Value (Lack of Fit)	Pure Error (Mean Square)
	X_1_	X_2_	X_1_* X_2_	X_1_^2^	X_2_^2^					
*Rc*	−5.85	7.63	−2.92	−12.40	−12.34	0.9899	0.9827	<0.0001	0.837	4.11
*F*-value	72.29	122.72	11.96	149.28	147.74	-	-	-	-	-
*p*-value	<0.0001	<0.0001	0.011	<0.0001	<0.0001	-	-	-	-	-

X_1_ = Pre-freezing temperature (°C); X_2_ = Sublimation temperature (°C).

## Data Availability

Not applicable.
